# Detection of Thrombi in Extracorporeal Membrane Oxygenation Circuits Using Low-Frequency Acoustic Measurements

**DOI:** 10.1097/MAT.0000000000002661

**Published:** 2026-02-10

**Authors:** Sheran Nanayakkara, Gabriel Fuchs, Marcus Brynolf, Lisa Prahl Wittberg, Lars Mikael Broman

**Affiliations:** From the * FLOW, Department of Engineering Mechanics, Royal Institute of Technology, KTH, Stockholm, Sweden; † Department of Anaesthesia, Surgery and Intensive Care, Sundsvall Hospital, Region Västernorrland, Sundsvall, Sweden; ‡ Department of Physiology and Pharmacology, Karolinska Institutet, Stockholm, Sweden; § ECMO Centre Karolinska, Pediatric Perioperative Medicine and Intensive Care, Karolinska University Hospital, Stockholm, Sweden.

**Keywords:** thrombi, infra-sound

## Abstract

Extracorporeal membrane oxygenation (ECMO) provides temporary support for patients with severe acute cardiac or respiratory failure. Although thrombotic complications are common during ECMO, the real-time detection and management of thrombosis-related complications remain challenging. In this study, we introduce a noninvasive approach for detecting thrombi using an acoustic detection technique. The presence of blood clots in the ECMO circuit has been correlated with an increase in the amplitude of the acoustic spectrum *<*20 Hz. This amplification allows for the prediction of clots in the ECMO circuit using acoustic measurements. The predicted results were compared with visual observations, and the technique achieved a total accuracy of 84%. Hypothesizing that some of the false positives were indicators of clots in the circuitry, the accuracy may be up to 96%. This technique could be integrated with the existing clot detection method to enhance patient outcomes.

## Graphical abstract

**Figure:**
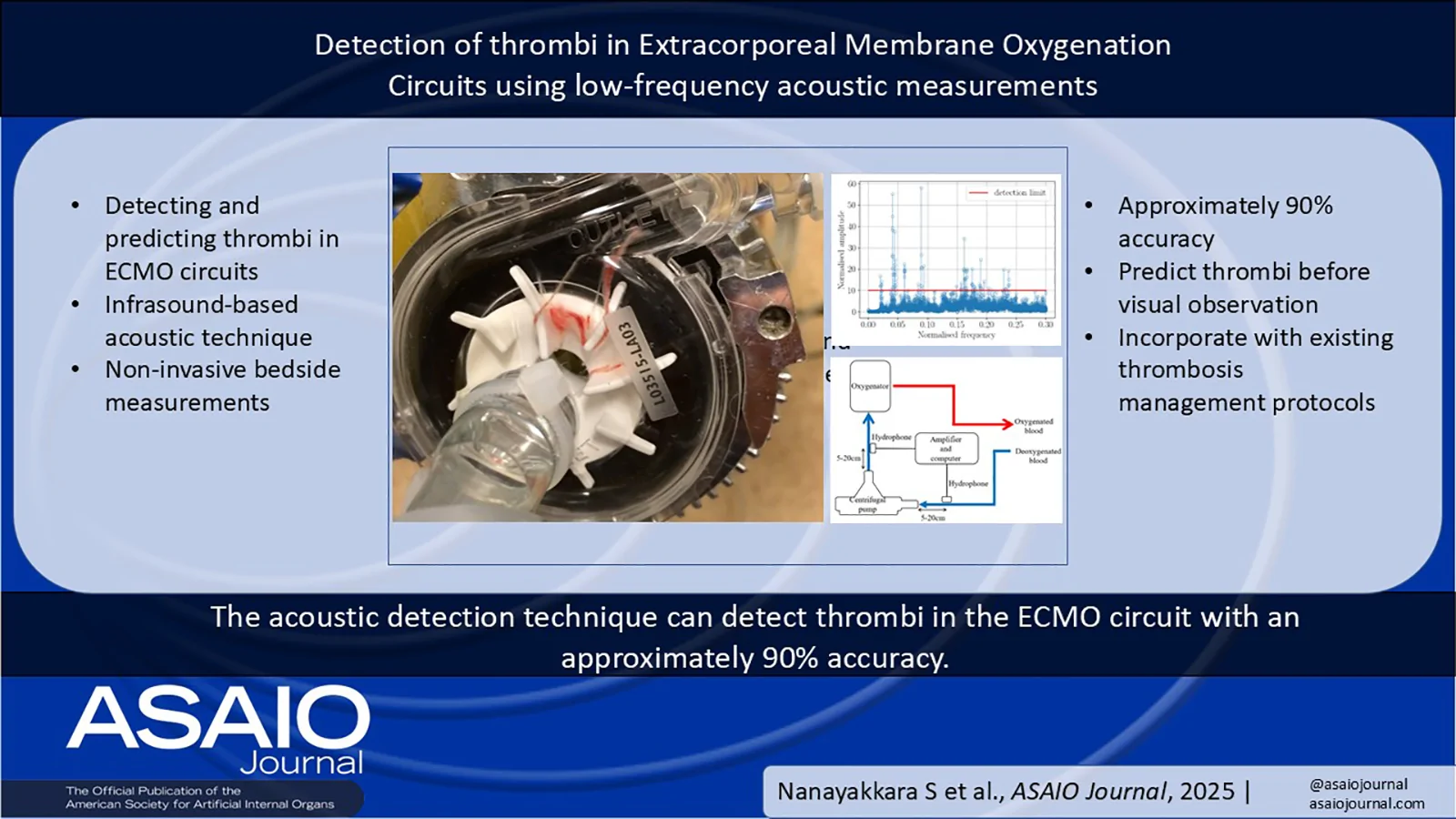

Extracorporeal membrane oxygenation (ECMO) is a lifesaving treatment for refractory acute cardiac and/or respiratory failure, allowing time to treat underlying conditions.^1^ Despite advances, thrombosis remains a significant concern due to interactions between blood and artificial circuit components and nonphysiological flow conditions promoting coagulation activation.^2,3^

Despite the high incidence of thrombus formation in ECMO circuits, real-time detection and management of thrombosis-related complications remain challenging. Typically, thrombus detection in the ECMO circuit relies on clinical judgment, laboratory data, and visual inspection of the pump and tubing. A comprehensive review of thrombosis management techniques used in ECMO is available in the literature.^4^

Previous studies have investigated acoustic data for clinical decision-making in similar contexts.^5–7^ Changes in the acoustic signature of left ventricular assist devices (LVADs) have been noted during clot passage.^7–9^ In mock-loop studies with clot analogues, low-frequency amplification was observed, likely due to rotor imbalance and blockage effects.^6,10,11^

Our previous research has demonstrated that infrasound measurements (<20 Hz) at the inlet and outlet of the centrifugal pump in ECMO circuits can be used to detect blood clots and their fragments.^12^ The approach included analysis of the strength of infrasound modes, consistent infrasound data, and clinical observations, resulting in blood clot detection with no notable false positives or negatives. In this study, the proof of concept originally presented is extended to a larger group of patients with a day-by-day detailed analysis, presenting a thrombus detection technique based on the acoustic signature of bedside measurements.^12^ The predictions based on the low-frequency detection technique were compared with clinically observed blood clots in the ECMO circuit to validate the accuracy of the proposed technique.

## Methodology

Bedside measurements were obtained from 14 patients for a cumulative total of 57 days at our ECMO intensive care unit. Acoustic measurements were obtained in 2015, 2016, and 2020 for eight, three, and three patients, respectively. The centrifugal pump CentriMag (Thoratec, Pleasanton, CA) was used for adults, whereas the DP3 pump (Xenios AG, Heilbronn, Germany) was used for neonates. In addition to acoustic data, the pump revolutions per minute (rpm), and flow rate (L/min) were recorded.

Clinical differences among patients, including ECMO configurations, were not considered. The focus was only on acoustic signal analysis. Acoustic data were passively collected using hydrophones attached outside of the tubing of the ECMO circuit without interfering with the treatment. Figure 1 shows a schematic diagram of the indicative locations for acoustic data acquisition. Simultaneous measurements were obtained at the inlet and outlet of the centrifugal pumps, typically 5–20 cm away from the pump inlet or outlet.

**Figure 1.**
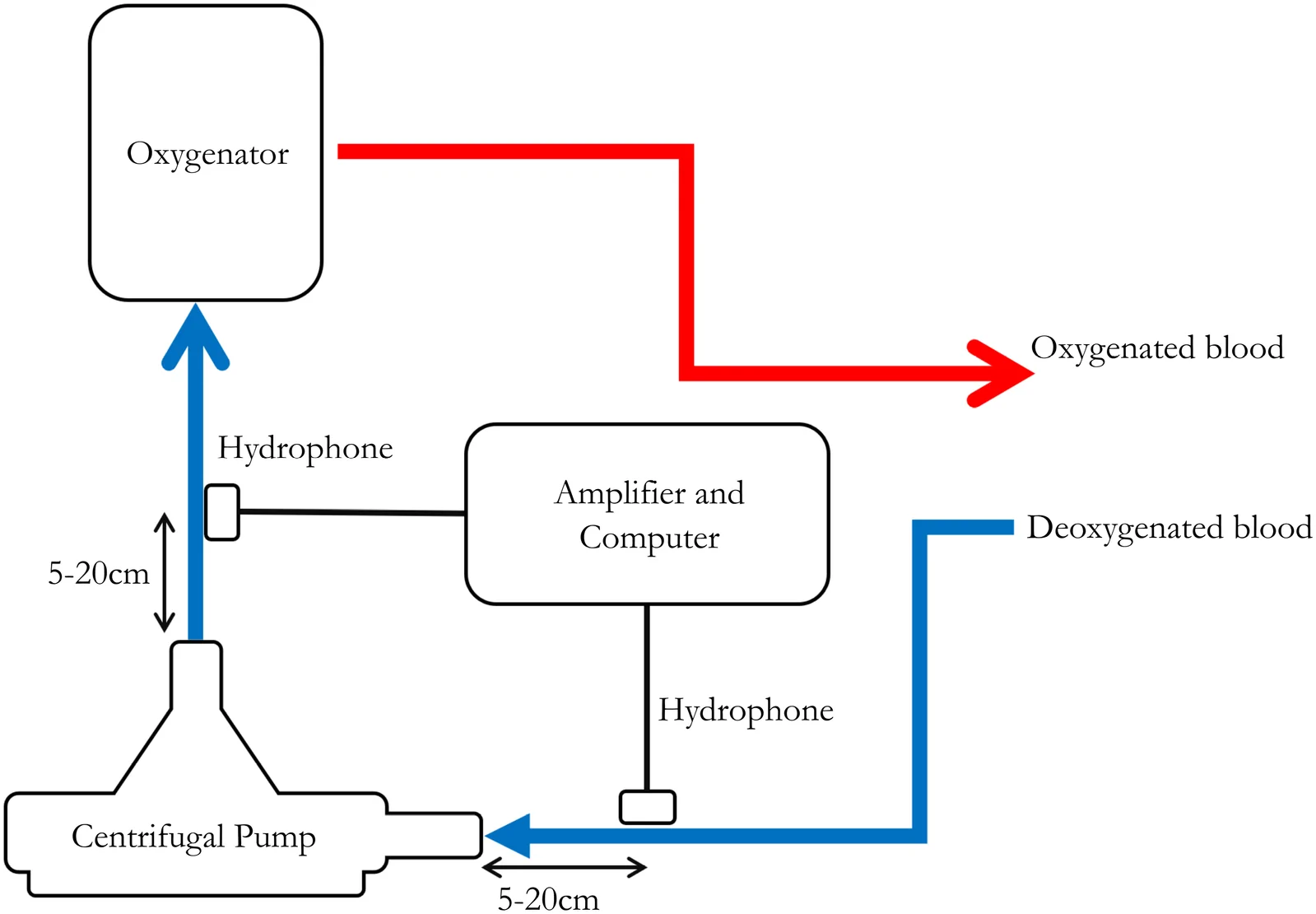
Schematic diagram of an ECMO setup indicating the location of acoustic measurements. ECMO, extracorporeal membrane oxygenation.

Additionally, the results from the regular inspections (three times daily) of each circuitry by the bedside staff were recorded. The tubing connected to and from the pump was inspected using a flashlight to illuminate and detect potential clots. Figure 2 shows two examples of blood clots found near the pump following cleansing after removal of the pump from a disconnected ECMO circuit.

**Figure 2.**
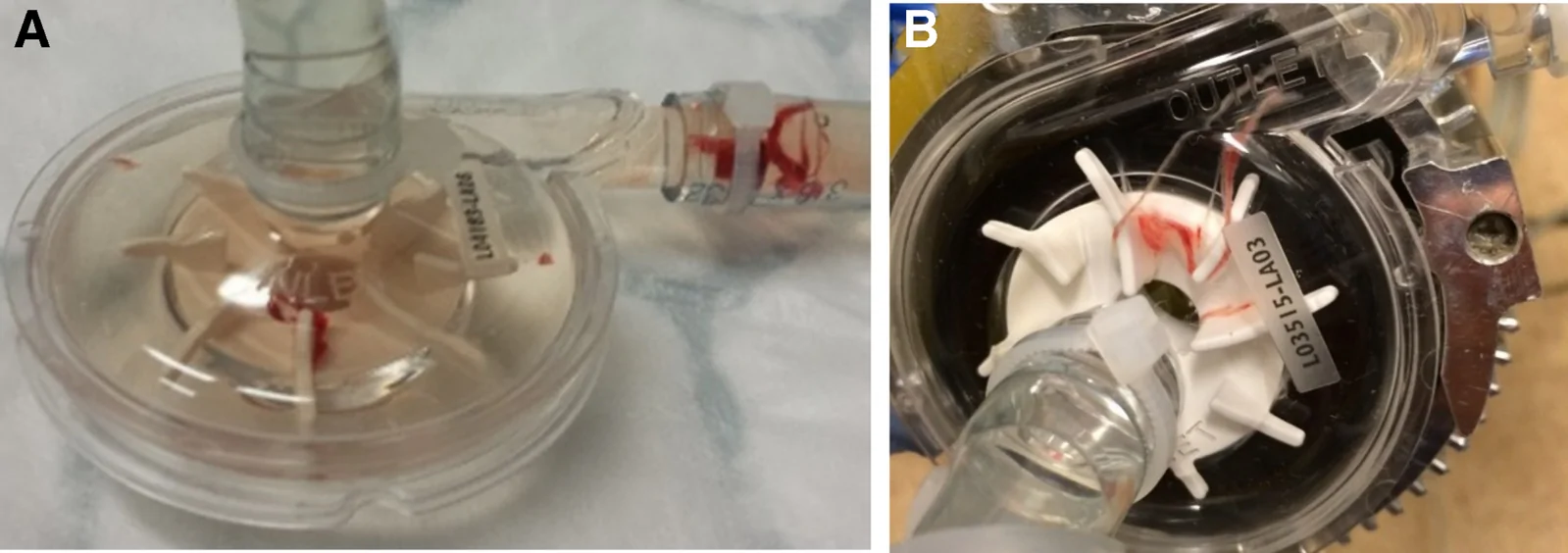
Examples of blood clots observed visually after the removal and careful cleansing of the ECMO circuit near the pump impeller and connection between pump outlet and tubing. ECMO, extracorporeal membrane oxygenation.

### Measurement Technique

Hydrophones (model 8103, Bruel & Kjaer), with a frequency range of 0.1 Hz to 180 kHz, were used to carry out the acoustic measurements. The frequency response of the hydrophone indicates a linear response to frequencies down to 2 Hz, showing reliable estimates of the low-frequency data. The hydrophone was connected to an amplifier and digitized at 44 kHz. Sampling times of 10, 30, and 60 seconds were evaluated. Longer sampling times (60 s) improved clarity in the noisy hospital environment, aiding signal analysis.

### Frequency Analysis: Identification of Low-Frequency Content

#### Signal analysis

First, the spectral content of the acoustic data was analyzed using a Fast Fourier Transform (FFT) technique using the Python environment.^13^ Generally, the largest peak in the FFT spectrum was the frequency corresponding to the pump speed (revolutions per minute, rpm).

#### Signal filtering

The calculated frequency spectrum was filtered using a low-pass filter. Frequencies less than 300 Hz were isolated.

#### Signal normalization

The filtered frequency spectrum of each acoustic measurement was scaled by the pump rotation frequency in both amplitude and frequency. As the pump rpm was determined by clinical requirements, it varied between each patient measurement. Generally, a constant rpm was maintained for each day of data collection. Therefore, this normalization was essential to compare the relative significance of the measured frequencies between days for the same patient and also between patients.

#### Comparison with reference signals

A reference signal was chosen as a representative signal of the circuit without any blood clots. The first two measurements of a set were typically chosen as reference signals. This reference and corresponding spectrum provided the baseline signal, to which the following measurements were compared. The calculated spectra for each patient on each day were divided by the corresponding reference signals to obtain the relative difference between the spectrum of interest and the reference signal. Figure 3 shows a spectrum at the pump inlet calculated using this approach. Here, only the <20 Hz (<0.3 normalized frequency) range is shown. Figure 3 shows that the measured acoustic signals were 10–60 times larger than the reference signal. For this specific case, blood clots were predicted and were also visually observed. The large peaks observed in this figure were typical of other cases where blood clots were predicted using this technique.

**Figure 3.**
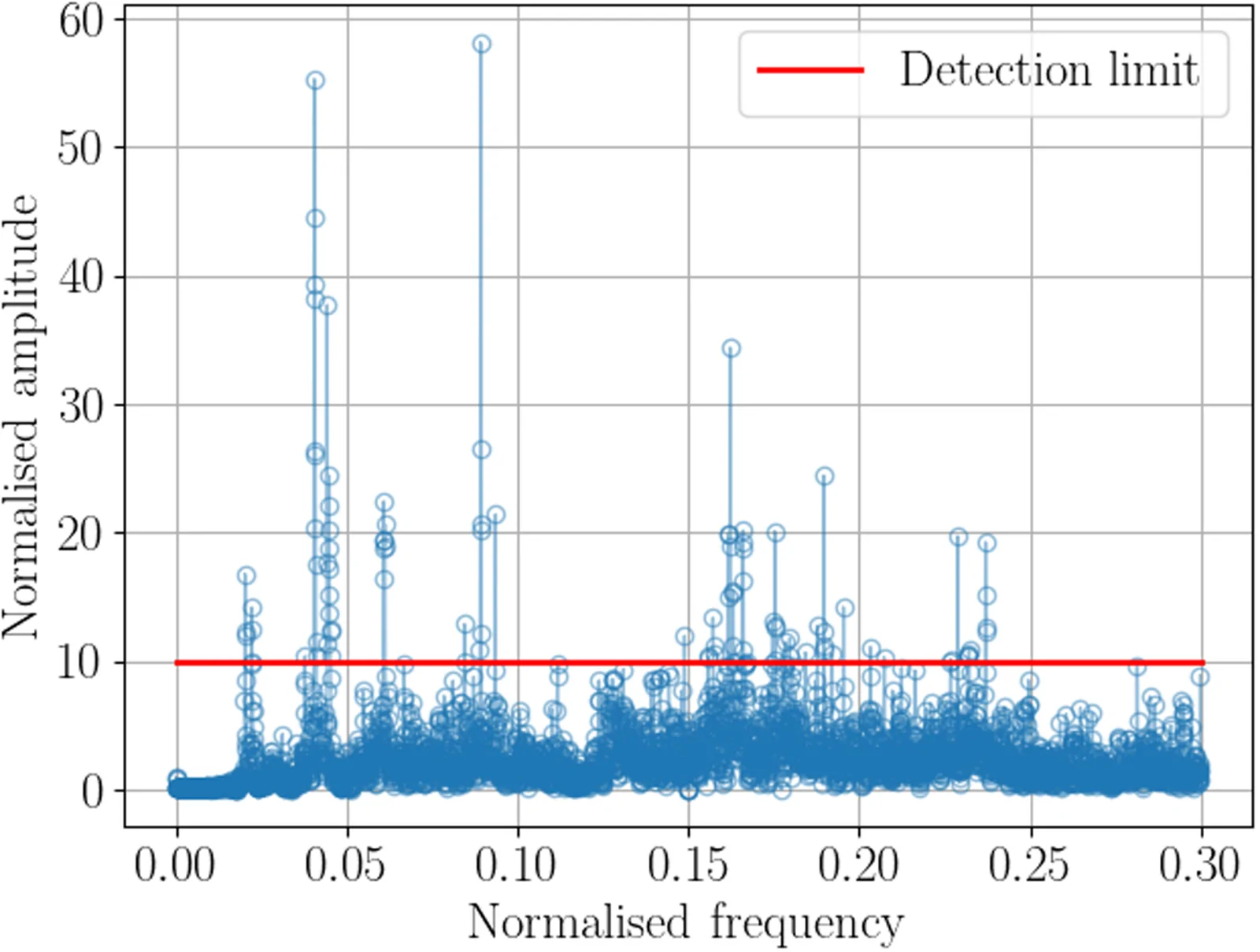
Normalized frequency spectrum.

#### Filtering using new parameters

Two new parameters were defined and denoted as detection limit and percentage limit, respectively. The detection limit serves as a threshold determining the significance of low-frequency spectrum amplification of the signal. In Figure 3, the solid red line represents a detection limit of “10,” indicating the frequency spectrum of the measured signal was 10 times larger than that of the reference signal. This parameter was empirically determined and may be adjusted to best fit the measured data. The number of points in the FFT spectrum above the detection limit (red line) was counted and expressed as a percentage of the total points, which is referred to as the percentage limit. In Figure 3, we observed approximately 21 peaks (with 93 points) above the red line, corresponding to 2.6% of points above the red line. If the threshold were set at 1%, then 2.6% of points above the red line would indicate the presence of a clot. A percentage limit of 0% will flag even a single peak above the detection limit as a positive thrombus detection, whereas a percentage limit of 50% requires broad-spectrum amplification within the <20 Hz range for a positive detection. At present, no specific frequencies or modes within the <20 Hz range have been identified as definitively amplified in the presence of blood clots. Thus, the percentage limit serves as a filter to characterize any amplification within this range as a potential indicator of blood clots. Additionally, the percentage limit provides a margin of error (*e.g*., 1%) to account for amplifications unrelated to thrombus formation in the ECMO circuit.

Together, these two parameters, the detection limit and percentage limit, were used to predict the presence of a clot in the ECMO circuit using measured acoustic signals. If either the signals from the inlet or the outlet met the criteria set above, a clot was predicted.

#### False predictions

A detection limit of 10 and a percentage limit of 1% were used to obtain the predictions. Supplemental Digital Content 1, https://links.lww.com/ASAIO/B841, provides a sensitivity study of these two parameters. In the case where a clot was predicted, but not clinically observed was referred to as “False positive.” “False negative” corresponded to instances when a blood clot was not predicted using the acoustic technique, although clinically observed.

## Results

The analyzed acoustic data obtained from the patients are summarized in Table 1. The table presents instances where clot presence was visually confirmed by the clinical team, alongside cases where the detection technique predicted a clot. The location where the clot was visually observed has also been noted. It also indicates whether each prediction corresponded with a confirmed visual observation, thereby distinguishing true positive and false positive predictions. The total accuracy of the technique was 84%. Thirteen false predictions were made, with two false negative predictions and seven false positive predictions. The total sensitivity was 84% while the specificity was 81%.

**Table 1.** Clot Observation and Prediction Details of Patients 1–14

Patient	Day	Observed (Location)	Predicted	False Prediction
P01	01	Yes (Pump Impeller)	Yes	-
P02	01	No	No	-
	02	Yes (Impeller and bottom of housing)	Yes	-
P03	01	No	No	-
	02	Yes (Pump outlet)	Yes	-
P04	01	No	No	-
	02	No	Yes	False positive
P05	01	No	No	-
	02	No	No	-
	03	No	No	-
P06 (neo)	01	Yes (Tubing)	Yes	-
	02	Yes (Pump outlet)	Yes	-
P07 (neo)	01	Yes (Pump Impeller)	Yes	-
P08 (neo)	01	Yes (Tubing)	Yes	-
P09	01	No	No	-
	02	No	No	-
P10	01	No	No	-
	02	No	No	-
	03	No	Yes	False positive
	04	No	No	-
	05	No	No	-
	06	No	No	-
	07	No	No	-
	08	No	No	-
P11	01	No	Yes	False positive
	02	Yes (Oxygenator)	Yes	-
	03	No	Yes	False positive
	04	No	No	-
	05	No	No	-
	06	No	No	-
	07	Yes (Oxygenator)	No	False negative
	08	No	No	-
	09	No	Yes	False positive
	10	No	No	-
	11	Yes (Oxygenator)	Yes	-
	12	No	Yes	False positive
	13	Yes (Connector)	Yes	-
	14	Yes (Oxygenator and pressure gauges)	Yes	-
P12	01	No	No	-
	02	No	No	-
	03	Yes (Oxygenator)	Yes	-
	04	Yes (Oxygenator and pressure gauges)	Yes	-
	05	Yes (Oxygenator and pressure gauges)	Yes	-
	06	Yes (Oxygenator and pressure gauges)	Yes	-
	07	Yes (Pressure gauges)	Yes	-
	08	Yes (Oxygenator)	Yes	-
	09	Yes (Connector)	Yes	-
P13	01	No	No	-
	02*	No	Yes	False positive
	03	Yes (Cannula drainage)	Yes	-
	04	Yes (Connector)	Yes	-
	05	Yes (Tubing)	Yes	-
	06	Yes (Tubing)	Yes	-
P14	01	Yes(Tubing)	Yes	-
	02	Yes (Tubing)	Yes	-
	03	Yes(Tubing)	Yes	-
	04†	Yes (Oxygenator)	No	False negative

* Pump change.

† Clots were removed from the circuit.

## Discussion

This article presents a method for detecting thrombosis in the ECMO circuit using an acoustic technique. The outlined methodology was capable of predicting the presence of circuit thrombosis with an 84% accuracy. The current technique for the identification of ECMO circuit thrombi is visual observation using a flashlight. The investigated technique may provide a continuous monitoring system for clinical staff that could act as a warning system for potential clots in the ECMO circuit.

The detection limit and percentage limit presented here are parameters fitted to match the measured data. Different values for these variables may result in different prediction levels. We expect, with more experimental measurements, that these variables may be tuned to be generally applicable for most cases. Additionally, these variables could be determined by clinical requirements. If suspecting circuit clots, clinicians may choose a lower detection limit to alert to potential clots in the circuit. Conversely, if a patient is characterized as having a low risk of thrombosis, limits may be increased to minimize the number of false alerts.

The false positives warrant further discussion. Since a simple method is used clinically to visually observe clots in the circuit (using a flashlight), it is possible that, in some instances, the false positives observed in Table 1 are early circuit thrombus indicators, not detected visually on the same day. For example, for patient 11, a clot was predicted on day 1, and clots were not visually observed. However, a clot was observed on day 2. A similar trend was seen for day 3, day 11, and further for patient 13 on day 02. These instances highlight that the proposed acoustic clot detection technique may be able to detect clots in the circuit before the clots can be visually observed or clots that are not clearly visible clinically.

Hypothesizing that some of the false positives were indicators of clots in the circuitry, and the false negatives were the true prediction errors, the accuracy of the method increases to 96%.

### Sensitivity to Reference Signals

An ECMO circuit is composed of many components and different tubing lengths, sensors, and other equipment connected to the circuit (*e.g*., dialysis machine). These may affect the measured reference signal or the acoustic signature without thrombi in the circuit. Therefore, the reference signal is crucial for a valid baseline acoustic signature. Moreover, the reference signal may vary from circuit to circuit. If any changes are made to the circuit (*e.g*., pump replacement or addition of equipment), a new baseline reference signal is needed, and all measurements from here on compared against this new reference.

### Potential Sources for the Low-Frequency Amplification

There are many other sources in proximity to the ECMO circuit which may influence its acoustic signature, arising from electrical, mechanical, or fluid sources. Since the present investigation was performed and data were obtained from patients in a clinical environment, isolation of acoustic sources was not possible.

It has been proposed that the source of the low-frequency amplification is from the interaction between the clot and the swirling vortex core generated by the centrifugal pump.^12^ This section explores the potential mechanisms that could lead to frequencies outside the typical range for a pump operating within its intended design parameters.

1. Rotor imbalance: A potential source of imbalance of the rotating pump is the presence of clots near the pump impeller (Figure 2).^10^ Similar observations were made for clot analogs, where an increase in amplitude was observed in the 1–10 Hz range.^11^

Furthermore, semistationary flow patterns developing under the impeller have been observed to cause the impeller to wobble in DP3 pumps, both numerically and experimentally.^14^ This wobbling behavior becomes more noticeable at reduced flow rates or elevated pump speeds. Therefore, rotor imbalance is a possible source of an acoustic signal distinct from the signal obtained for a clean circuit. However, the isolation of the frequencies amplified by this imbalance is presently unknown and warrants further investigation.

2. Flow instabilities connected to the flow in ECMO circuit components that may result in a low-frequency component and amplification thereof are several:
  - Frequency analysis of the flow structures obtained from computational fluid dynamics (CFD) simulations of blood pumps revealed peaks corresponding to both the blade passing frequency and the rotor rpm.^14^ A strong correlation between the blade passing frequency (a rotor with four blades has a blade passing frequency four times the pump rpm) and the turbulent kinetic energy of the fluid was observed. However, no significant peaks were detected in the spectra below 20 Hz. This finding suggests that the low-frequency amplification observed in the present study is not solely due to the fluid passage through the pump but rather results from the interaction of blood clots with the native fluid flow, an effect not considered in the CFD simulations.^14^
  - Flow instabilities within the pump, often caused by operating outside the best efficiency point, can lead to pressure pulsations and vibrations, typically associated with low-frequency components.
  - The potential accumulation of emboli in the helical vortex created by the centrifugal pump may also lead to a variation in the acoustic signal. It has been shown that heavy particles are attracted to helical-shaped trajectories,^15^ and get trapped in the central or outer region of the vortex by the balance between Stokes drag and centrifugal force, depending on the pitch of the helices. Therefore, it is likely that different distributions of particles near the wall or in the center of the tube will lead to different peaks in the measured acoustic signal.
  - Thin, long blood clots are generally observed at connectors between tubes and other components. Figure 2A shows an example of a blood clot at the pump outlet. Such clots are captured in the swirling jets exiting the centrifugal pump, and due to the added mass of the clots, they may excite frequencies lower than the pump rpm.
3. Blockages in the flow into or out of the pump can cause variations in the acoustic signature of the signal. These blockages may be a result of larger blood clots.
4. Resonance can occur if the natural frequencies of the pump’s structural elements or mounting system coincide with operational frequencies or harmonics, amplifying vibrations at those frequencies.
5. Cavitation: This is the formation and collapse of small vapor bubbles, which can generate vibrations. While cavitation is unlikely to occur under design conditions in centrifugal blood pumps, practical factors such as the use of clamps and tube blockages may create conditions that lead to cavitation in the pump. Cavitation in centrifugal pumps in ECMO circuits has been previously discussed.^16,17^
6. Viscoelastic effects: Another potential is the ratio of the clot’s viscoelastic modulus to the flow’s dynamic pressure, a mechanism analogous to wind-induced vibrations in a flag. The vibration frequency of a thrombus depends on its shear modulus, length, blood density, and flow velocity.

Furthermore, the method proposed herein is independent of the type of pump or ECMO configuration used.^10^ Since the reference signals are obtained for each specific case, that is, for a specific pump or configuration, and corresponding results are compared against the same reference signal, the type of pump or configuration does not affect the outcome of the predictions.

The acoustic measurements may be influenced by the preload and afterload of the ventricles. However, the amplitude of these pressure fluctuations is expected to be insignificant compared to the amplitude of the pump rotations. Furthermore, the frequencies of preload and afterload pressure changes are likely to be within the 0.5–1 Hz region, and thus, in the lower end of the measurement range of interest of the current technique. Considering these aspects, the current measurements are expected to be independent of preload and afterload conditions.

### Limitations and Recommendations for Future Study

We have identified certain limitations in the present study and recommend the following for future study.

1. Acoustic measurements: The present acoustic measurements were obtained using B&K hydrophones with a nominal operating range between 0.1 and 180 kHz. However, when approaching the <2 Hz, the accuracy of the device may decrease, as such devices are typically designed to measure higher frequencies. Therefore, the use of a device that has a design operation range that includes a <2 Hz range, such as an accelerometer, is recommended. Furthermore, longer acoustic measurements or continuous measurements will likely improve the accuracy of predictions.
2. Reference signals: The reference signals used for the present investigation were selected from the available measurements from data without any visual clots observed. We recommend that these reference signals be obtained in-clinic shortly after implantation of ECMO to ensure that a clean circuit signal is effectively captured.
3. Additional measurement locations: Obtaining acoustic measurements from more than two locations may assist in identifying the location of the clot in the circuit.
4. CFD analysis: Multiphase CFD analysis of the pump with clot analogs inserted into the circuit will provide useful information on the coupling between the fluid-solid behavior.
5. Another possible percentage limit may be defined by comparing the acoustic energy levels stronger than a threshold as compared to the total acoustic energy level in the low-frequency range (1–20 Hz).
6. Investigation of clot analogs in a mock circulation loop, together with measurement of the associated acoustic signal amplifications, will provide valuable insights.
7. The relationship between thrombus size and signal amplitude needs to be evaluated to improve the method’s specificity further.

## Conclusions

We present a method for detecting thrombi in ECMO circuits using an acoustic detection technique. The proposed approach is noninvasive and does not require any modifications to the ECMO circuit. Based on a 10-fold amplification of the measured signal compared with a reference signal, clot predictions were obtained. The predicted results were compared against the visual observation, and the proposed technique yielded a total accuracy of 84%. Excluding false positives, the accuracy of the technique increased to 96%. Rotor imbalance due to larger blood clots, the swirling motion of smaller and longer clots in the helical flow patterns at the pump inlet and outlet, or tubing, and viscoelastic effects are likely causes of the observed acoustic amplification. The technique proposed here may be used to develop a simple system to warn clinicians of potential blood clots in the ECMO circuit. Such a device, coupled with clinical observations, may significantly improve the efficacy. This would provide clinicians with an additional indicator to further investigate the circuit, complementing current practice and potentially enhancing the early detection of thrombus formation. Using the knowledge gained from the investigation and the technique proposed, we plan to conduct further detailed bedside measurements for validation of the method in ECMO patients. The preliminary results presented here indicate that the technique is suitable for thrombus detection. However, further *in vitro* and *in silico* validation is required to improve the sensitivity and specificity of the method.

## Acknowledgments

Funding from the Swedish Heart Lung Foundation (Hjärt-Lungfonden) and Departmental research funding from Region Stockholm are greatly acknowledged.

## Supplementary Material

## References

[1] Thiagarajan RR Barbaro RP Rycus PT; ELSO member centers: Extracorporeal Life Support Organization Registry International Report 2016. ASAIO J 63: 60–67, 2017. 10.1097/MAT.0000000000000475 27984321

[2] Cooper E Burns J Retter A: Prevalence of venous thrombosis following venovenous extracorporeal membrane oxygenation in patients with severe respiratory failure. Crit Care Med 43: e581–e584, 2015. 10.1097/CCM.0000000000001277 26308437

[3] Fuchs G Berg N Broman LM Prahl Wittberg L: Flow-induced platelet activation in components of the extracorporeal membrane oxygenation circuit. Sci Rep 8: 13985, 2018. 10.1038/s41598-018-32247-y PMC6143512 30228350

[4] Zeibi Shirejini S Carberry J McQuilten ZK Burrell AJC Gregory SD Hagemeyer CE: Current and future strategies to monitor and manage coagulation in ECMO patients. Thromb J 21: 11, 2023. 10.1186/s12959-023-00452-z PMC9878987 36703184

[5] Slaughter MS Feldman CM Sobieski MA: Utilization of acoustic signatures to identify HeartMate XVE device end-of-life. J Heart Lung Transplant 26: 579–583, 2007. 10.1016/j.healun.2007.03.009 17543780

[6] Nakazawa T Makinouchi K Takami Y Glueck J Tayama E Nose Y: Vibration assessment for thrombus formation in the centrifugal pump. Artif Organs 21: 318–322, 1997. 10.1111/j.1525-1594.1997.tb00368.x 9096805

[7] Kaufmann F Hormandinger C Stepanenko A: Acoustic spectral analysis for determining pump thrombosis in rotary blood pumps. ASAIO J 60: 502–507, 2014. 10.1097/MAT.0000000000000097 24814834

[8] Hubbert L Sundbom P Loebe M Peterzen B Granfeldt H Ahn H: Acoustic analysis of a mechanical circulatory support. Artif Organs 38: 593–598, 2014. 10.1111/aor.12244 PMC4209799 24372095

[9] Inan OT Baran Pouyan M Javaid AQ: Novel wearable seismocardiography and machine learning algorithms can assess clinical status of heart failure patients. Circ Heart Fail 11: e004313, 2018. 10.1161/CIRCHEARTFAILURE.117.004313 PMC5769154 29330154

[10] Kawahito K: Transformation of vibration signals in rotary blood pumps: the diagnostic potential of pump failure. J Artif Organs 16: 393–396, 2013. 10.1007/s10047-013-0709-1 23625149

[11] Cairelli AG. *Improvement of a non-invasive method for clot detection in ecmo circuit and localization of thrombotic events*. Politecnico di Milano; 2019.

[12] Fuchs G Berg N Eriksson A Prahl Wittberg L: Detection of thrombosis in the extracorporeal membrane oxygenation circuit by infrasound: Proof of concept. Artif Organs 41: 573–579, 2017. 10.1111/aor.12782 PMC5484328 27654663

[13] Harris CR Millman KJ van der Walt SJ: Array programming with NumPy. Nature 585: 357–362, 2020. 10.1038/s41586-020-2649-2 PMC7759461 32939066

[14] Nilsson F Broman LM Prahl Wittberg L: Canonical flow structures formed in a diagonal pump used in extracorporeal membrane oxygenation. Phys Fluids 37: 061903, 2025.

[15] IJzermans RHA Hagmeijer R van Langen PJ: Accumulation of heavy particles around a helical vortex filament. Phys Fluids 19: 107102, 2007.

[16] Lequier L Horton SB McMullan DM Bartlett RH: Extracorporeal membrane oxygenation circuitry. Pediatr Crit Care Med 14: S7–S12, 2013. 10.1097/PCC.0b013e318292dd10 PMC3742331 23735989

[17] Pedersen TH Videm V Svennevig JL: Extracorporeal membrane oxygenation using a centrifugal pump and a servo regulator to prevent negative inlet pressure. Ann Thorac Surg 63: 1333–1339, 1997. 10.1016/s0003-4975(97)00098-2 9146324

