# Supplementary material for: Detection of Thrombi in Extracorporeal Membrane Oxygenation Circuits Using Low-Frequency Acoustic Measurements
Source: ASAIO J. 2026 Feb 10;72(11):e348–54. doi: 10.1097/MAT.0000000000002661 (PMC13704694; doi:10.1097/MAT.0000000000002661)
Supplement: Supplementary file 1 [file mat-72-e348-s001.pdf]

Supplementary Material

Based on the methodology discussed in the manuscript, 14 patients were investigated over a cumulative total of 57 days. The predictions were sensitive to the two key parameters, *detection limit* and *percentage limit*, and this sensitivity is discussed in detail here. False positives are instances where blood clots are predicted but not clinically observed, while false negatives are instances when blood clots were not predicted using the acoustic technique but were clinically observed. Total false predictions are the sum of both false positive and false negative instances. The aim was to identify the combination of parameters that minimises both total false predictions and false negatives.

Firstly, a suitable detection limit was chosen. Two different percentage limits were tested, and the impact of changing the detection limit on prediction accuracy was studied in Figure 1. The detection limit ranged from 1 to 50. It was observed that false negatives increased with increasing detection limit, while false positives decreased. When the detection limit was very small (approximately 1–2) or very large (approximately 30–50), a larger total false prediction rate (around 45%) was observed at both percentage limits. Therefore, detection limits that are too small or too large increase prediction error and are not suitable. A detection limit between 5 and 10 provided the best balance, minimising false predictions while keeping false negatives low.

Next, the effect of the percentage limit on predictions was examined in Figure 2 while keeping the detection limit constant at 5 and 10. Increasing the percentage limit led to an increase in false negative predictions. A detection limit of 10 combined with a small percentage limit (1%) minimised total false predictions while keeping false negatives low. These parameter values were therefore chosen for further detailed analysis.

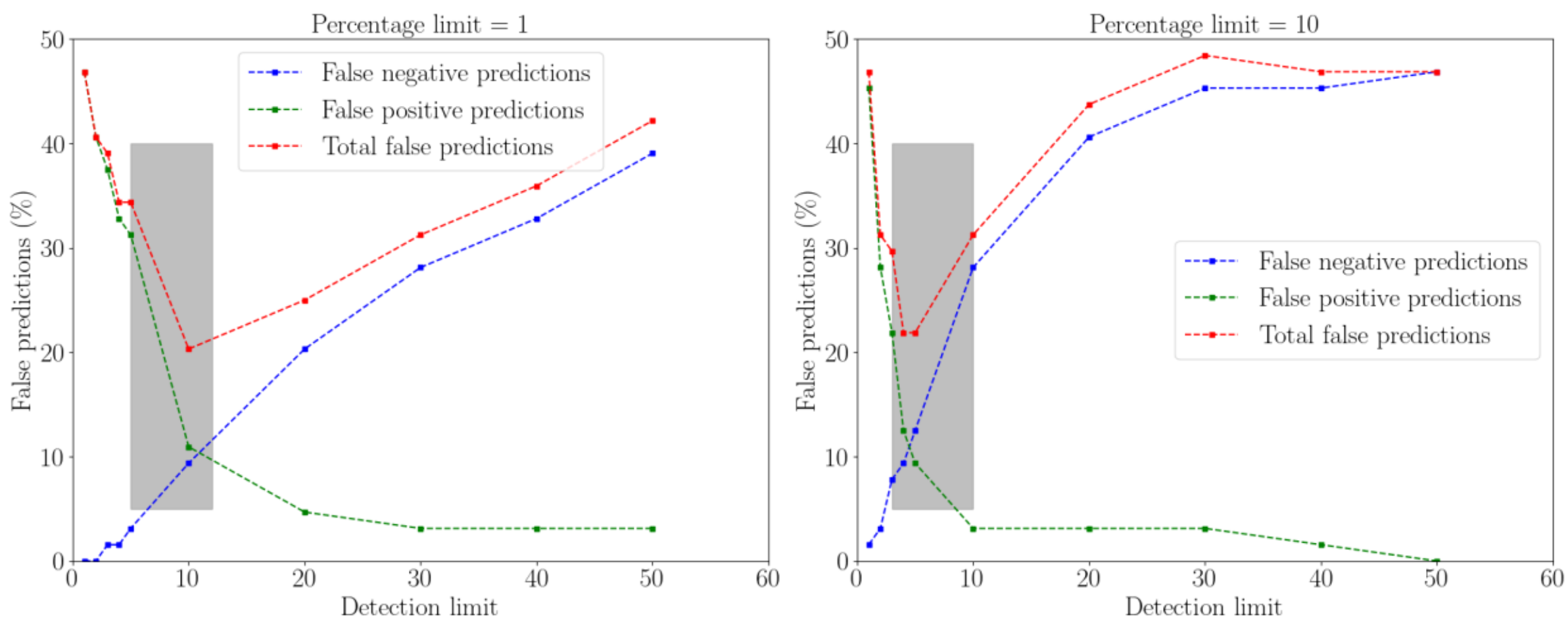

Figure 1: Variation of detection limit for fixed percentage limit

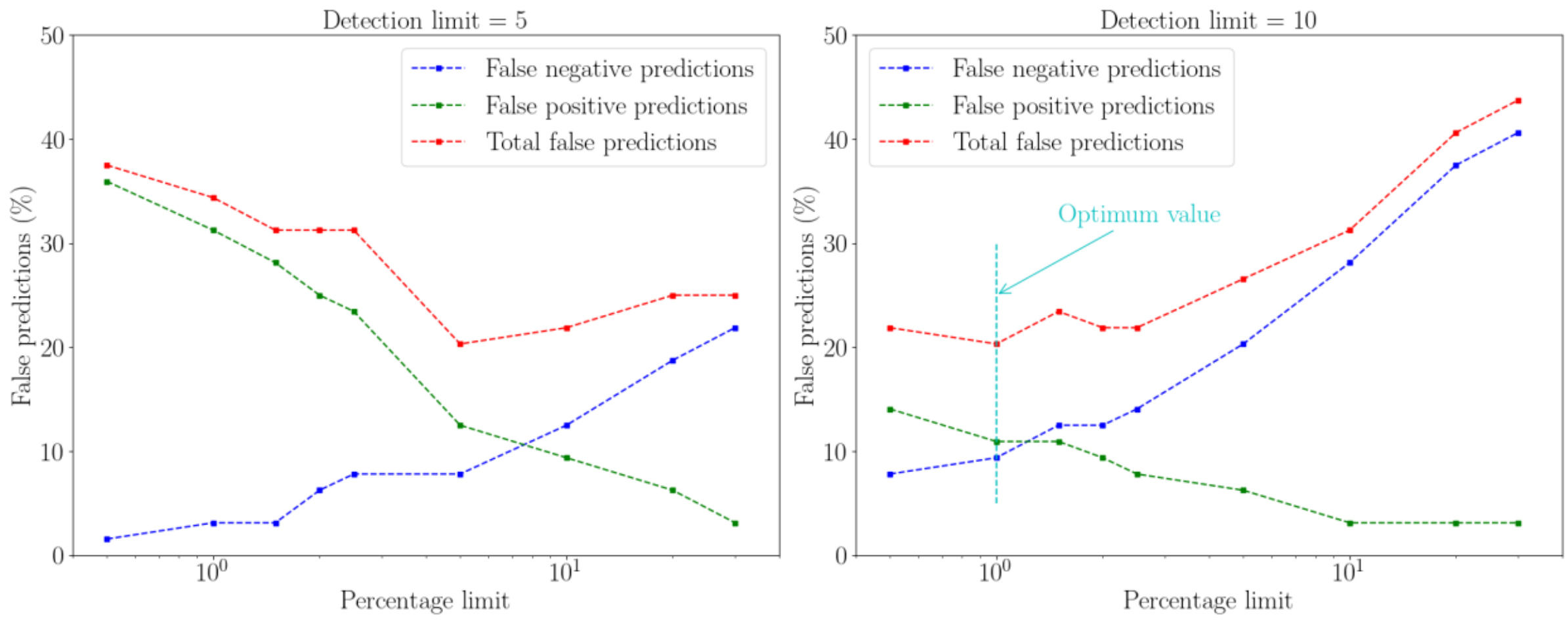

Figure 2: Variation of percentage limit for fixed detection limit
